# Ultrasonographic evaluation of the supraspinous ligament in a series of ridden and unridden horses and horses with unrelated back pathology

**DOI:** 10.1186/1746-6148-3-3

**Published:** 2007-03-01

**Authors:** Frances MD Henson, Luis Lamas, Sabina Knezevic, Leo B Jeffcott

**Affiliations:** 1 Department of Veterinary Medicine, University of Cambridge, Madingley Road, Cambridge CB3 0ES, UK; 2 Faculty of Veterinary Science, University of Sydney, NSW 2006, Australia

## Abstract

**Background:**

Injury to the supraspinous ligament (SSL) is reported to cause back pain in the horse. The diagnosis is based on clinical examination and confirmed by ultrasonographic examination. The ultrasonographic appearance of the supraspinous ligament has been well described, but there are few studies that correlate ultrasonographic findings with clinical pain and/or pathology. This preliminary study aims to test the hypothesis that unridden horses (n = 13) have a significantly reduced frequency of occurrence of ultrasonographic changes of the SSL consistent with a diagnosis of desmitis when compared to ridden horses (n = 13) and those with clinical signs of back pain (n = 13).

**Results:**

The supraspinous ligament of all horses was imaged between T(thoracic)6-T18 and ultrasonographic appearance. There was an average of 2.08 abnormal images per horse from the whole group. The average number of abnormalities in unridden horses was 4.92, in ridden horses 2.92 and in horses with clinical back pain 4.69. No lesions were found between T6 and T10 and 68% of lesions were found between T14 and T17. No significant difference (p < 0.05) was found between the three groups in the number or location of abnormal images.

**Conclusion:**

The main conclusion was that every horse in this study (n = 39) had at least one site of SSL desmitis (range 2 to 11). It was clear that ultrasonographically diagnosed SSL desmitis cannot be considered as *prima facie *evidence of clinically significant disease and further evidence is required for a definitive diagnosis.

## Background

Back pain is a common presentation in equine practice and one that can arise from a variety of conditions, involving both soft tissue and vertebral damage. Soft tissue pathology encountered in the equine back includes muscular and ligamentous damage, and of the different clinical conditions reported, supraspinous ligament (SSL) desmitis has been implicated as a cause of clinical back pain by a number of authors [2,4,7].

The SSL is the extension of the nuchal ligament in the thoracolumbar region. The SSL runs along the summits of the dorsal spinous processes (DSPs) of the thoracic and lumbar vertebrae to its terminal insertion on L5, forming attachments at each summit. It receives multiple tendon insertions of *longissimus dorsi *that contribute to its structure[5]. Injury to this ligament may occur as a result of direct trauma, but it is considered to be an athletic injury due to overstretching of ligament fibres, particularly when jumping[4]. Diagnostic imaging of the SSL is performed using ultrasonography [2,3].

The ultrasonographic appearance of the SSL has been described by Denoix [2] and Gillis [4]. In the withers region the ligament is wide and the presence of secondary centres of ossification of T6 – 9 may case acoustic shadows into the ligament. As the ligament descends into the thoracic and lumbar regions the ligament becomes larger and more echogeneic, whilst in the caudal thoracic and lumbar regions obliquity of the longitudinal fibres as they attach over the apex of the dorsal spinous processes can affect the image. At all sites the SSL appears more hypoechogeneic as it courses over the interspinous space. In addition to these observations on the static anatomy of the ligament it has been suggested that when the SSL is relaxed (e.g. during extension of the thoracolumbar areas), vertical hypoechoic images may be induced by the lack of tension in the ligamentous fibres.

A number of ultrasonographic changes have been described as indicative of SSL desmitis[2,4]. These include hypoechoic images (indicative of acute desmopathy), changes to the fibre pattern, hyperechoic images (indicative of chronic desmopathy) and core lesions. However, there remain a number of important questions in relation to the accurate interpretation of the ultrasound findings in the clinical setting. These include the variability in the normal echogenic pattern of the SSL and the lack of suitable reference data in horses which exhibit no apparent signs of back pathology. In addition, the authors' clinical experience indicates that ultrasonographic lesions in the SSL are a relatively common finding in horses presented for unrelated clinical conditions. In order to ascertain the significance of ultrasonographic changes within the SSL we consider it important to examine the correlation between ultrasound changes and the use of the horse (i.e. ridden or unridden) and the presence of clinical back pain. In this preliminary study we hypothesised that unridden horses would have a significantly reduced frequency of occurrence of ultrasonographic changes of the SSL consistent with a diagnosis of desmitis when compared to ridden horses and those with signs of back pain. The results of the study show that there is no significant difference in the ultrasonographic appearance of the SSL between these groups and that ultrasonographic lesions of the SSL cannot be considered as *prima facie *evidence of clinically significant disease.

## Results

### General findings and their location

**Figure 1:**
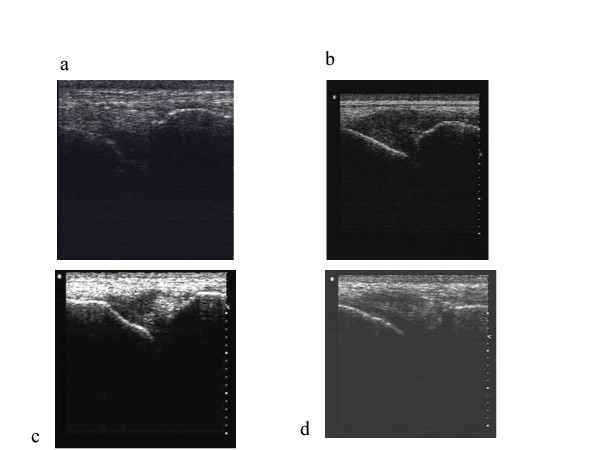
Longitudinal ultrasonograms of the supraspinous ligament. (a) Normal appearance of the supraspinous ligament (b) A representative hypoechogeneic lesion (c) A representative hyperechogeneic lesion (d) A representative alteration of supraspinous ligament fibre pattern.

There were a total of 163 abnormal findings identified in the SSL of the 39 horses in this study. These lesions included alterations in fibre pattern, hypoechogeneic lesions and hypoechogeneic lesions (Figure 1). This comprised an average of 2.08 (SD 1.72) abnormal images per horse, with 99 (61%) located in the ligament as it traversed the interspinous space and 64 (39%) above the DSPs (Table 1). A Tukey's-HSD test demonstrated that no two groups were significantly different at a p < 0.05 level.

The total number of abnormal images in Group 1 was 64 with an average of 4.92 (SD 2.01) per horse. The location of these images showed that 39 (61%) of them were in the interspinous space and 25 (39%) above the summits of the DSPs (Table 2)

In Group 2 there was a total of 38 abnormal images with an average of 2.92 (SD 2.10) per horse with 31 (82%) located in the interspinous space and 7 (18%) above the DSPs. While in Group 3 there were a total of 61 abnormal images and an average of 4.69 (SD 2.17) per horse with 29 (48%) located in the interspinous space and 32 (52%) above the DSPs.

A one-way ANOVA of abnormal images above the summits of the DSPs indicated a highly significant difference (p = 0.0009) with Group 2 having significantly fewer abnormal images. However, a one-way ANOVA of abnormal images located in the interspinous spaces indicated there was no significant difference (p = 0.44) between the groups.

**Figure 2:**
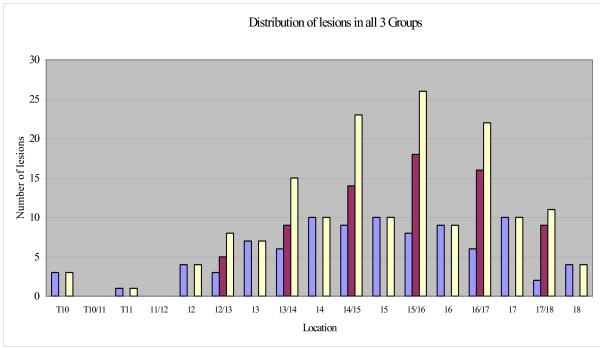
A graph to show the distribution of abnormal images of the supraspinous ligament in the 39 horses studied.

No ultrasonographic abnormalities were found in any of the groups between T6 and T10 and the majority of the findings were present in the area between T14 and T17 (26% of the total number of locations scanned), which accounted for 111 (68%) of the total number of abnormalities (Figure 2).

### Hypoechoic images

A total of 92 (56%) hypoechoic images were identified with an average of 2.35 (SD 1.8) per horse when all three groups were combined, 30 (33%) were located in interspinous space and 62 (67%) above the summits of the DSPs. Horses in Group 1 (unridden) were found to have an average of 2.38 (SD 1.8) hypoechoic areas per horse, with 6 (19%) located in the interspinous space and 25 (81%) above the summits of the DSPs.

Group 2 was found to have 1.61 (SD 1.6) hypoechoic images per horse, 14 (66%) of them located in the interspinous space and 7 (34%) above the summits of the DSPs.

Group 3 had 3.07 (SD 1.8) hypoechoic areas per horse 10 (25%) of them in the interspinous space and 30 (75%) above the summits of the DSPs. No significant differences were found between the groups in the prevalence of hypoechoic abnormal images (p = 0.12).

### Hyperechoic images

A total of 71 (44%) hyperechoic images with an average of 1.82 (SD 1.6) per horse were identified in this study, 69 (97%) abnormal images were found in the interspinous space and 2 (3%) above the summits of the DSPs. Group 1 had an average of 2.53 (SD 1.80) hyperechoic areas per horse and all 33 (100%) of them were present in the interspinous space.

Group 2 had 1.30 (SD 1.43) abnormal images per horse and Group 3 had 1.61 (SD 1.38) abnormal images per horse. All 17 of these images were located in the interspinous space in Group 2, and in Group 3 only 2/19 lesions (approximately 10%) were detected in this location. No significant differences were found between Groups in the incidences of hyperechoic abnormal images (p = 0.125).

## Discussion

Ultrasonographic examination of the SSL has become a routine step in the diagnostic work up of cases with back pain [10]. The diagnosis of SSL desmitis has been established when there was a loss or increase in the normal echogenicity of the ligament, including core lesions, and/or a loss or disruption of the parallel fibre pattern of the ligament[4]. However, due to the change in direction of the fibres, normal variations of echogenicity can occur. These include hyperechoic images in the withers region which cast acoustic shadows, a general increase in echogenicity in the caudal thoracic region, vertical hypoechoic images that may be induced by the relaxation of the ligament, and apparent hypoechogenicity of the interspinous portion of the ligament [2,10]. In this study images found outside these descriptions were considered as abnormal, as has been described by Denoix [2] and Gillis [4].

The first conclusion that can be drawn from this study is that every horse included in this study (n = 39) had at least one site of SSL desmitis (range 2 to 11). Therefore it is clear that ultrasonographically diagnosed SSL desmitis cannot be considered as *prima facie *evidence of clinically significant disease. Given that the average number of abnormal images per horse in this study was 2.08, it was important to identify whether there were any differences between ridden and unridden horses, and then between these groups and horses with clinically significant back pain. As SSL desmitis causes back pain it might have been expected that lesions of SSL desmitis might be over-represented in the horses with back pain included in this study (included with no case selection in linear sequence of admission to the clinic). A Tukey's test demonstrated that there was no significant difference in the distribution of abnormal images in the three different groups. However, the one-way ANOVA did show a significant difference in the number of abnormal images in the ridden horses in Group 2, compared to those unridden and horses with clinical back pain i.e. they had significantly less lesions. If this finding had been solely in relation to horses with clinical back pain then it may be of interest, however, the fact that it was also less than in unridden horses complicates the interpretation. This finding does not, at least, support the hypothesis that riding and athletic activity causes lesions consistent with a diagnosis of SSLD, as has been suggested previously (Gillis 1999).

Whilst abnormal images were recognised in all Groups of horses, abnormal images were not evenly distributed throughout the entire thoracolumbar spine. Most abnormal images were detected between T14 and T17, which agrees well with what has previously been described[7]. No ultrasonographic changes were seen between T6 and T10 in any group of animal, indicating perhaps the strength of the origin of this ligament. The paucity of lesions at this site would suggest that SSL desmitis is genuinely rate at this site and abnormalities at this site may be more significant than abnormalities at other sites, should they be demonstrated. However, one limitation of this study is that it did not include a rigorous investigation of the repeatability of the diagnosis of pathology of the SSL using ultrasonography and this area requires further investigation.

The overall number of SSL abnormalities and their type and site were analysed. It was shown that hypoechoic abnormal images were more common in this series of horses than hyperechoic (mean 2.35 per horse vs 1.82 per horse). There were no significant differences between groups of horses in the numbers of either hyperechoic or hypoechoic abnormal images, although it was noted that 97% of hyperechoic images were detected in the interspinous spaces. Hyperechoic lesions *not *in the interspinous space may therefore be of more clinical significance. It is difficult from these results to identify which types of ultrasound changes are most clinically significant or in what numbers they become significant as they were relatively evenly distributed between horses.

## Conclusion

Abnormal images of the SSL have been associated with pain [2,4,10] and SSL desmitis has been made on the basis of detecting hyperechogeneic and/or hypoechogeneic abnormal images. However, it appears to have been assumed that, as in the case of other ligaments in the body such as the suspensory ligament, ultrasonographic changes detected in a ligament are strongly suggestive of underlying pathology or injury. An important observation from this study is that abnormal ultrasonographic images were detected in all groups of horses, regardless of whether or not the horse had been ridden or exhibited clinical signs of back pain. The average number of abnormal images per horse was 2.08, with 61% of these occurring in the interspinous space and 39% occurring above the DSPS. It can be concluded, therefore, that the presence of a lesion in the SSL may occur with no other evidence of clinical disease. In order to prove that these lesions cause pain it is strongly recommended that local anaesthesia techniques should be used as an adjunct to diagnosis, although the proximity of the DSP and the effects of solution diffusion may make interpretation difficult.

The clinical importance of potentially abnormal ultrasound findings can only be evaluated when combined with a clinical history, physical examination, local analgesic techniques and the absence of other diagnostic evidence that might explain the origin of the pain.

## Methods

### Subjects

A total of 39 horses referred to The Queen's Veterinary School Hospital were used and were divided into three groups:

▪ Group 1: 13 horses, not worked or ridden previously, that on clinical examination revealed no signs of any back pain. The average age of this group was 6.2 years (SD = 3).

▪ Group 2: 13 horses, used regularly as general riding horses, but with no previous history of any clinically back related problem and no signs of back pain on clinical examination. The average age of this group was 10.4 years (SD = 6.2). Horses were selected prospectively for inclusion in the study from the start date of the study, but no other case selection was practised.

▪ Group 3: 13 horses, used regularly as general riding horses that had been diagnosed with back pain on clinical examination. The average age of this group was 8.3 years (SD = 2.3). Cases were selected prospectively for inclusion in the study from the start date of the study, but no other case selection was practised.

### Evaluation of back pain

A detailed clinical history for all horses was obtained, focussing on details of possible back pathology [6,7]. A clinical examination with the horse in stocks was performed that consisted of visual inspection for assessment of conformation and symmetry of the hindquarters and back muscles. Palpation of the back was carried out to detect any signs of muscle tension or areas of pain particularly involving the dorsal spinous processes (DSPs) and surrounding epaxial structures. Manipulation of the thoracolumbar spine was performed to elicit the range of flexion and extension in the sagittal plane (spinal dorsiflexion, ventroflexion) and lateral flexion [6]. This was followed by examination of the horse's gait and performance in hand and on the lunge in a soft sand ménage. Horse in groups 2 and 3 were also evaluated at ridden exercise as part of our standard protocol.

### Ultrasonographic examination

All horses were finely clipped over the DSPs extending from T6 caudally to T18, the area was cleansed with chlorhexidine and alcohol, and then coated with aqueous contact transmission gel. Ultrasonographic evaluation of the SSL was performed using a linear and a sector array transducer with frequencies between 5 and 10 Mhz [1,2] (Figure 1a). Images were obtainable with both types of scanner and at all frequencies. However the linear scanner at 7.5 Mhz gave the most detailed pictures in the opinion of these authors. Longitudinal and cross sectional views were obtained of the ligament both in the areas over the DSP summits and the interspinous spaces.

### Image classification

Lesions were considered as abnormal if any of the following changes were identified[2,4]; hypoechoic images (Figure 1b), hyperechoic images (Figure 1c), core lesions and detectable changes in fibre pattern (Figure 1d). The location of the abnormal images was recorded as being above the named DSPs or in the interspinous space.

### Statistical analysis

Statistical analysis was carried out using a one way ANOVA with the Post-Hoc analysis done using the Tukey's Honestly Significant Difference test (Tukey's-HSD test) to compare the findings of the three groups.

## Abbreviations

DSIL – Dorsal sacro-iliac ligament

SSL – Supraspinous ligament

## Authors' contributions

1. FMDH designed the study, performed the pilot studies and helped to draft the manuscript.

2. SK carried out the ultrasonography.

3. LL carried out analysis and interpretation of the data and drafted the manuscript.

4. LBJ clinically examined the cases and helped to draft the manuscript. All authors read and approved the final manuscript.

## Figures and Tables

**Table 1:** The total numbers, means and standard deviations of ultrasonographic abnormalities of the supraspinous ligament in the three different groups of horses studied.

**Groups**	**Hypoechoic**	**Mean (SD)**	**Hyperechoic**	**Mean (SD)**	**Total**	**Mean (SD)**
**G1 (Unridden)**	31	2.38 (1.8)	33	2.53 (1.80)	64	4.92 (2.01)
**G2 (No pain)**	21	1.61 (1.60)	17	1.30 (1.43)	38	2.92 (2.1)
**G3 (Pain)**	40	3.07 (1.8)	21	1.61 (1.38)	61	4.69 (2.17)
**Total**	92	2.35 (1.81)	71	1.82 (1.60)	163	4.17 (2.23)

SD = standard deviation

**Table 2:** The total numbers of of hypoechogeneic and hyperechogeneic lesions at different anatomical sites in the three groups of horses studied.

**Groups**	**Interspinous space**		**Above DSP**		**Total**
		
	**Hypoechoic**	**Hyperechoic**	**Hypoechoic**	**Hyperechoic**	
**G1(Unridden)**	6	33	25	0	64
**G2 (No pain)**	14	17	7	0	38
**G3 (Pain)**	10	19	30	2	61
**Total**	30	69	62	2	163
